# Microfluidic enrichment for the single cell analysis of circulating tumor cells

**DOI:** 10.1038/srep22076

**Published:** 2016-02-29

**Authors:** Trifanny Yeo, Swee Jin Tan, Chew Leng Lim, Dawn Ping Xi Lau, Yong Wei Chua, Sai Sakktee Krisna, Gopal Iyer, Gek San Tan, Tony Kiat Hon Lim, Daniel S.W. Tan, Wan-Teck Lim, Chwee Teck Lim

**Affiliations:** 1 Clearbridge Accelerator Pte Ltd, 81 Science Park Drive, The Chadwick, #02-03, Singapore Science Park 1, Singapore 118257, Singapore; 2 School of Biological Science, National Technological University, 60 Nanyang Drive, Singapore 637551, Singapore; 3 Cancer Therapeutics Research Laboratory, National Cancer Centre Singapore, 11 Hospital Drive, Singapore 169610, Singapore; 4 Department of Pathology, Singapore General Hospital, Outram Road, Singapore 169608, Singapore; 5 Cancer Stem Cell Biology, Genome Institute of Singapore, 60 Biopolis St, #02-01, 138672, Singapore; 6 Division of Medical Oncology, National Cancer Centre Singapore, 11 Hospital Drive, Singapore 169610, Singapore; 7 Duke-NUS Medical School, 8 College Road, 169857, Singapore; 8 Institute of Molecular and Cell Biology, A*Star, 61 Biopolis Drive Proteos, 138673, Singapore; 9 Department of Biomedical Engineering, National University of Singapore, 4 Engineering Drive 3, Block E4, #04-08, Singapore 117583, Singapore; 10 Mechanobiology Institute of Singapore, 5A Engineering Drive 1, Singapore 117411, Singapore

## Abstract

Resistance to drug therapy is a major concern in cancer treatment. To probe clones resistant to chemotherapy, the current approach is to conduct pooled cell analysis. However, this can yield false negative outcomes, especially when we are analyzing a rare number of circulating tumor cells (CTCs) among an abundance of other cell types. Here, we develop a microfluidic device that is able to perform high throughput, selective picking and isolation of single CTC to 100% purity from a larger population of other cells. This microfluidic device can effectively separate the very rare CTCs from blood samples from as few as 1 in 20,000 white blood cells. We first demonstrate isolation of pure tumor cells from a mixed population and track variations of acquired T790M mutations before and after drug treatment using a model PC9 cell line. With clinical CTC samples, we then show that the isolated single CTCs are representative of dominant EGFR mutations such as T790M and L858R found in the primary tumor. With this single cell recovery device, we can potentially implement personalized treatment not only through detecting genetic aberrations at the single cell level, but also through tracking such changes during an anticancer therapy.

Traditional biological cell assays normally measure the contents of entire sample population, thus neglecting intercellular variations^1^. Cell to cell variability has been observed in cells even within the same culture^2^,^3^, and can manifest as differences in genomic expressions^4^, cell cycle stages^5^ and cellular responses when exposed to an environmental stimuli^6^. Emerging data is beginning to highlight the complexity of cancer and its clinical relevance. With a deeper understanding of intra-tumor and inter-cellular heterogeneity, it is apparent that traditional sequencing methodologies – where cellular information is averaged – is an under-representation of the biological complexity^7^,^8^,^9^,^10^. Drug resistance remains a pervasive challenge, and recent efforts have been directed at characterizing mechanisms in order to devise novel therapeutic strategies^11^,^12^,^13^,^14^. Serial sampling is typically required to examine dynamic changes temporally^15^,^16^. Traditional biopsies which are invasive, are difficult to acquire repeatedly over an extended time period^17^. Furthermore, intra-tumoral heterogeneity presents challenges in obtaining a complete profile of the disease^18^,^19^,^20^. Circulating tumor cells (CTCs) which represent hematogenous dissemination from the solid tumors is a viable option^21^. These cells can potentially form secondary metastases and hold important evidences that can account for disease progression^22^,^23^.

Challenges that exist in CTC analyses primarily lie in the excessive amounts of accompanying white blood cells (WBCs) in whole blood^24^,^25^. A substantial number of microfluidic based CTC enrichment systems have been developed that aims to provide reliable CTC detection and analysis. Platforms that are based on antibody affinity^26^,^27^,^28^, size based separation^29^,^30^ and flow based assays^31^,^32^ have achieved relatively good success in CTC detection and analysis. Despite cancer cell recovery rates as high as 95%, contaminating WBCs in the background remain an issue for downstream molecular analysis^33^. The background WBCs can hinder various downstream molecular assays with its abundant copies of wild-type DNA. This results in mutant signatures being marginalized in pooled CTC sample studies. The analysis is further complicated by the fact that CTCs are themselves heterogeneous^34^,^35^ and low frequency mutations of interest will be obscured without a very sensitive downstream assay. For example, in a clinical trial that detected EGFR mutations in non-small cell lung cancer (NSCLC) patients, Punnoose *et al*.^36^ had low concordance rates for CTCs with 1 in 8 samples matching the corresponding primary tumor. A similar study conducted by Marchetti *et al*.^37^ however, showed a remarkable concordance rate of 94% in 31 cases with matching tissue samples. The improvement was attributed to the starting DNA templates which had higher tumor cell concentration and using a sensitive readout with ultra deep sequencing. It is imperative that the CTC counts are high if the assays are to be meaningful. In one study conducted by Maheswaran *et al*.^38^, the median number of captured cells was 74 cells/ml and they achieved a high matching data of 92%. However, CTC counts vary in different patient profiles which pose problems for its clinical adoption. Hence, the application of single cell analysis, which represents pure tumor contents for disease diagnosis and monitoring, will further enhance the sensitivity of the assay.

In order to retrieve these rare single CTCs from enriched blood samples, contaminating WBCs will have to be discarded. As the absolute CTC number is considered small, this poses problems for conventional methodologies involving fluorescence activated cell sorters (FACS) due to its inherent system losses. Although it had been successfully applied to isolate single CTCs, the cell loss varied considerably with different studies^39^,^40^. Other approaches which had been successful in recovering single CTCs included micropipette aspiration^41^,^42^ and laser microdissection^43^ to manually “pick” these cells one by one. Although the results have been promising, the extremely low throughput and tedious nature of the procedure renders these methods difficult to implement in a clinical setting. Notable commercial platforms for single cell capture include the DEPArray from Silicon Biosystems and C1 from Fluidigm. The former which sorts more than ten thousands cells at the same time is a useful tool but suffers from sample losses of approximately 40% in one study conducted on CTCs^44^. The latter system, Fluidigm C1 is a fully integrated tool to prepare genomic samples from single cells with high fidelity. Some of the major drawbacks include limited capacity and it requires a homogeneous sample input. Recent advances in microfluidic technologies provided many advantages for single cell analysis such as increased sensitivity and economies of scale due to small volume reactions. Various systems demonstrated the capabilities to even integrate genetic library preparation on chip^45^,^46^,^47^, and provide large scale sorting using magnetic separation^48^ and DEP^42^. Single cell analysis had also been performed *in situ* with careful culture conditions replicated on devices^49^,^50^.

Here, we describe a novel microfluidic device capable of high throughput specific selection and isolation of single rare cells within a mixed cell population. This device utilizes hydrodynamic focusing to restrict cells in the flow and passively hold them in active control chambers alongside the main channel. By combining both passive and active elements, we are able to quickly and efficiently trap single cells and yet have the flexibility to select and separate any cell or cells of interest. As proof of principle, we recovered single cells from CTC samples via WBCs depletion on the device and correlated EGFR mutations to its primary tumor molecular characteristics. Using Sanger sequencing, we validated the ability to detect two different mutations (L858R and T790M) in the EGFR gene, associated with TKI response and resistance, respectively. With these clinical samples, we further demonstrated the efficacy for retrieval of small numbers of CTC from a background of approximately 20,000 cells. Our results showed strong concordance with the primary analyses done on tumor biopsies. This device has the potential to realize single cell analysis of CTCs for the clinical monitoring of cancer by not only enabling the capture of any specific CTCs of interest, but also with 100% purity.

## Results

### System workflow and working principle

**Figure 1:**
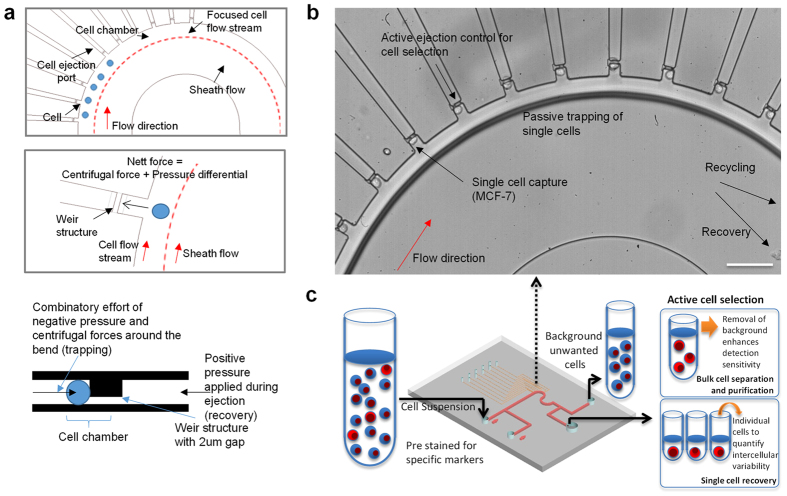
Demonstration of microfluidic single cell capture and recovery. (**a**) Schematic illustration of cell capture principle. Hydrodynamic focusing of cell flow stream by sheath flow. Cell preferentially enters a chamber due to a nett force towards the chamber (**b**) Device with isolated MCF-7 cells, a breast adenocarcinoma cell line in each of the chambers. The scale bar represents 100 μm. (**c**) Experimental workflow and device capabilities to enhance detection and allow for single cell analysis.

A schematic of the chip design is shown in Fig. 1a. This device utilizes hydrodynamic focusing with the help of a viscous sheath flow buffer which focuses the cells entering the device into a single cell stream. The cells are then ushered into the holding chambers due to the inherent differential pressure at these chambers. These cell chambers are lined along the outer curvature of the channel so that cells traversing through it will experience a slight centrifugal force that facilitate their entry into these chambers. The design of each chamber consists of a weir structure which increases in fluidic resistance when occupied. Once a cell enters the chamber, the fluid flow is blocked in that region thereby directing the cell stream to the other available chambers. For cell recovery, a positive pressure is exerted through a particular chamber to push the selected cell back into the main stream and into a collection port which will be opened.

More specifically, the layout of the device consists of a total of ten chambers which can be further expanded to accommodate more cells for higher throughput. Each chamber is attached to a dedicated control line that provides positive pressure during the cell recovery phase. When deactivated, they are exposed to atmospheric conditions. Figure 1b shows a working demonstration for single cell capture of MCF-7 cells. Cells are captured in the passive mode and the depth of the chambers ensures only one cell enters. Any potential pile up of cells is washed off in the main cell flow to the next available chamber. Each of the cells can then be ejected sequentially so that they can be individually recovered at the collection well. By incorporating a selection mechanism such as tagging antibody markers by immunofluorescence staining, we can achieve cell separation in mixed populations. In order to enhance the recovery of target cells, we recycle the effluent from the first pass of cell suspension back into the system. This ensures we maximize the yield from each sample and minimize any losses.

We envision the device to aid in enhancing sample/disease detection with active selection of single cells of interest as noted in Fig. 1c. The device is flexible to pool together the pure target cell population or recover single target cells for analysis. Achieving perfect separation of mixed cell populations is especially important in many different applications such as for CTC analysis. The pure tumor cell output will resolve various biomolecular assay issues, mainly limited by the excessive amounts of wild-type DNA. This in turn increases the sensitivity for detecting mutations in each sample. As a proof of concept, we recovered single tumor cells from enriched cancer blood samples and matched point mutations on these CTCs with mutations identified on the bulk tumor. Target cells are immediately removed from the device as each cell ejection sequence is activated. Single cell analysis of the sample will allow us to track and quantify key mutations within the CTC pool with fine resolution. With these cells, we aim to track key mutations within EGFR that are related to drug resistance in NSCLC.

### Device optimization via hydrodynamic focusing

**Figure 2:**
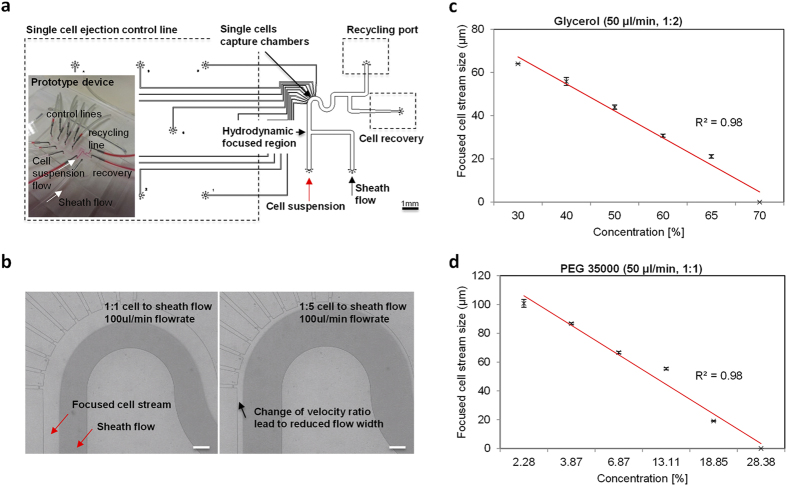
Device characterization. (**a**) Design representation showing cells enter via the cell flow, are hydrodynamically cornered by a sheath flow and are directed towards the 10 isolation chambers. Active selection based on immunofluorescence staining and/or morphology of cells decides whether they will be ejected into the recovery or recycling port. Scale bar represents 1 mm. (**b**) Illustrations of different flow rates of cell to sheath flow from 1:1 to 1:5 on how the cell flow width can be controlled. The scale bar is 100 μm. (**c**) Effects of using different concentrations of Glycerol in optimizing cell flow widths. (**d**) Effects of using different concentrations of PEG in optimizing cell flow widths. Data is denoted as Mean ± SEM.

Our approach to efficient single cell isolation is to restrict cells in suspension to its curved region as shown in Fig. 2a. This ensures the cell chambers are in close proximity and facilitate capture. A viscous flow buffer is used in a crossflow channel so as to focus the cell flow stream to the outer side. The use of a higher viscosity liquid allows the volume of the buffer used to be minimized while achieving optimal cell flow width. Due to higher viscosity in the sheath buffer, lower flowrates are required to achieve the desired larger sheath flow width in the microdevice. Ideally, the dimension of the cell flow stream should match the size of a typical cell to ensure the cells flow in a single file near the capture chambers. A larger than desired width will result in higher losses and reduce capture efficiency. Considering this aspect, we aim to achieve a 20–25 μm cell flow width.

We have tested various concentrations of glycerol and polyethene glycol (PEG) to achieve our desired flow dimension. Both buffers are biologically compatible^51^,^52^ and have demonstrated numerous biological applications such as aiding in drug delivery and as an antibacterial agent. Controlling either the sheath to cell flowrates or increasing the concentration of the buffers can attain the focusing effect. As illustrated in Fig. 2b with colour dyes, the cell flow width in the fluidic channel decreases from 100 μm to 20 μm with a five times increase in flow velocities. The relationship is linear (data not shown) and can be controlled in a straightforward fashion. Also under steady state condition, we observed a distinct line of separation between the two flows. This ensures cells remain in the cell flow stream and will not cross over into the sheath buffer during operations.

Alternative to increasing the velocity ratio of the two input flows, the change in viscosity ratio has the same effect. With increasing viscosities of the sheath buffer as shown in Fig. 2c,d, the cell flow width can be adjusted to the desired dimensions. The relationship is linear as well, with increasing sheath buffer viscosity leading to lower cell flow sizes. In using glycerol, we are able to maintain stable flow conditions with a flowrate ratio of 1:2 for cell to sheath flows (Fig. 2c) and 1:1 using PEG as the buffer (Fig. 2d). The R^2^ for using the two different buffers with varying concentrations are both 0.98, highlighting the ease in tuning the flow width. The benefit of changing the sheath buffer concentration is in its operational ease to minimize waste generation from each sample processed. It is expected the waste generated is roughly equal to the volume of the input cell suspension using PEG. We anticipate samples of fairly large volumes from CTC enriched specimens which is in the range of 5–10 ml and will be able to process these samples with minimal preparations. This reduces the chance of losses due to sample preparation or transfer when using our device. We are able to realize the desired cell flow conditions with a cell flowrate of 50 μl/min using glycerol at 65% and PEG at 19% as shown in the calibration data Fig. 2c,d.

### Single cell capture efficiency and demonstration of pure tumor cell separation from a mixed population

With the desired operating conditions, we performed single cell capture efficiency measurements on the device. Polystyrene beads of monodispersed sizes of 20 μm and 15 μm in diameters were used. Flow conditions were maintained at 50 μl/min with 65% glycerol used as the sheath buffer. We actively measured the time taken to fill all 10 cell chambers with particles of varying sizes to simulate the biological cell samples. Filling of cell chambers were fairly straightforward once the flow reached steady state as shown in movie S1. Cells entered the chambers sequentially and once occupied remained so until the ejection mode was activated. The only instance where cells missed a chamber was when large debris entered and disrupted the steady state flow between the sheath and cell flow. As our main channel is large, the debris can be effectively carried to waste without clogging the device and cell capturing event will then return to normal.

**Figure 3:**
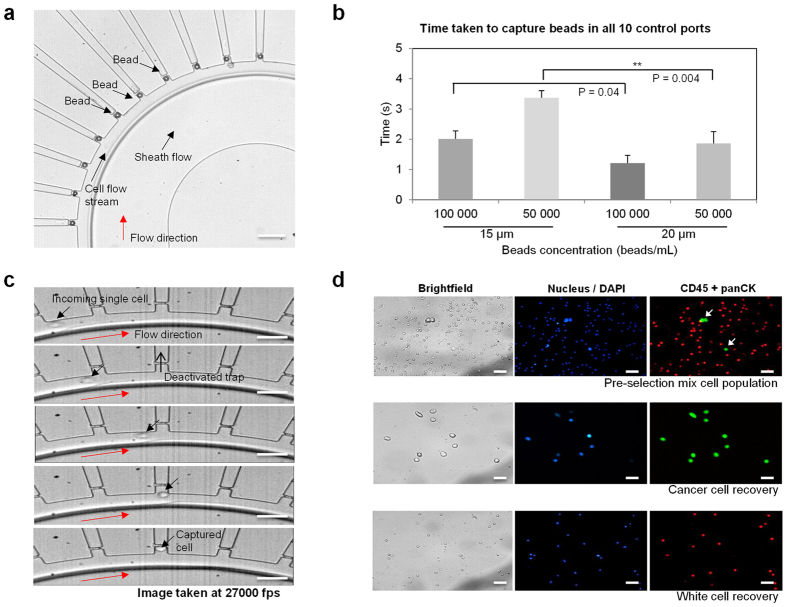
Capture efficiency and cell separation. (**a**) Demonstration of polystyrene beads (20 μm) in capture chambers. Scale bar is 100 μm. (**b**) Capture efficiency using 50,000 beads/ml and 100,000 beads/ml. Error bars represents standard error tabulated from 10 independent measurements. p value less than 0.01 are deemed statistically significant. (**c**) Process of cell capture acquired at high speed of 27 000 fps. Scale bar represents 50 μm. (**d**) Immuno-fluorescent images of spiked MCF-7 cell line in WBCs under 20x magnification before and after the selection process. The scale bar represents 50 μm.

Figure 3a shows the state of a fully loaded system with beads after 3 seconds of flow. We ejected all the particles in the chambers and resumed the flow process for another reading. An average of 10 measurements were taken for each sample condition as tabulated in Fig. 3b. We varied the bead concentration between 50,000 and 100,000 beads per milliliter of bead suspension. This ensures the system can handle different sample conditions. At 100,000 beads per milliliter, we were able to load the device within 1.61 sec and for 50,000 beads per milliliter of bead suspension, within 2.61 seconds. Unloading of beads from the chambers was instantaneous once each of the control fluidic lines was activated with a positive pressure. Using a Student’s t-test, there was no significant difference in time taken for beads of different sizes with input concentration of 100,000 beads/ml (*p* value of 0.04) and a nominal decrease in time (*p* value of 0.004) at lower concentrations of 50,000 beads/ml. These results show that we were able to isolate and retrieve the cells quickly and with ease.

We next focused on cell separation from a predefined mixed population. Having determined the optimal operating conditions and the rate of capture, we applied a control spiked sample into the system for processing. MCF-7 cells spiked in WBCs in the ratios of 1:1000 to 1:100,000 cells were used, thus simulating a blood sample that underwent primary enrichment for CTCs. Figure 3c shows a high speed capture of a MCF-7 cell into a deactivated cell chamber. We observed no difference between capturing cells or beads, and the chambers were able to hold a single cell until release. During cell ejection, the main flow was stopped and the recycling port closed. The recovery port was opened and the fluidic control line was activated as demonstrated in movie S2. In order to identify and recover the target cell population within the mixed group, positive selection using fluorescence markers like cytokeratin (CK) to label cancer cells or negative depletion of hematopoietic cells with CD45 markers can be used. Positive selection allows the study of cancer cell subtypes of interest that might be present in peripheral blood and negative depletion maximizes the yield of non-blood cells present in blood. For visualization, we stained both CK and CD45 markers. The samples were then processed on the device as illustrated in supplementary Fig. 1. For all the cells that were CK positive, they were directed towards the recovery port. All other cells were pushed towards the recycling outlet. Figure 3d shows the state before and after separation. By actively depleting WBCs shown in red, we can greatly enhanced the purity of the tumor sample. The active selection ensures only target cells remain to achieve perfect separation. The platform successfully brought together all cancer cells into a single collection well while depleting WBCs in the process. It takes roughly 2–3 hrs to process the sample of about 101,000 cells.

**Figure 4:**
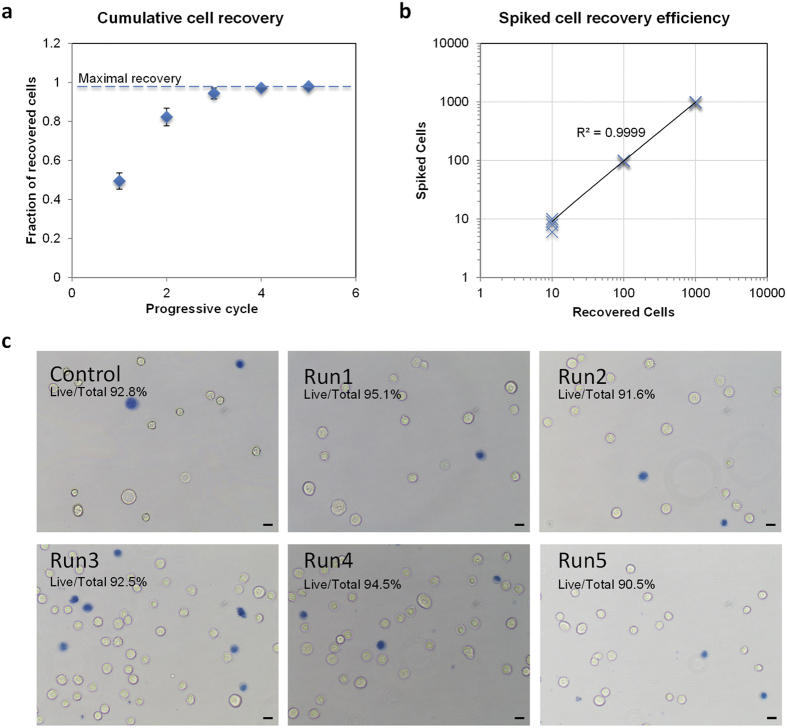
Efficiency for spiked cell recovery using MCF-7 cells showing minimal cell losses and cell damage from the processing of samples in the device. (**a**) Recovery efficiency with additional processing cycles with the trend leveling after cycle 3. Each data point was from an average of 15 independent runs. (**b**) Efficiency after cycle 3 for different spiked input conditions. (**c**) Live dead assay performed on cells processed from the device.

On average, it is observed that we require the effluent to be recycled three times for optimal cell recovery. To ascertain the cell losses, we measured the cell retrieved after every cycle for all 3 different spiked conditions as noted in Fig. 4a. Each spiked condition was independently performed five times. Overall, we observed relatively good cell recovery at every state with efficiencies greater than 95% after three recycle processing of the effluent. The return after cycle 5 was close to 99%. As the curve plateaus out after cycle 3, we recommend at least three processing cycles be done on each sample. Figure 4b illustrates the recovery efficiency after cycle 3 for all 15 runs. For spiked conditions of 100 and 1000 cells, we registered mean retrievals of greater than 98%. Details of recovery at every processing cycle are provided in supplementary Fig. 2. The recovery efficiencies ranged from 99–99.3%, 97–99% and 90–100% for spiked cell conditions of 1000, 100 and 10 MCF-7 cells. We envisioned each CTC sample will have to be put through the isolation process three times, and confident that the cell losses from each sample will be minimal.

For assessing the extent of cell damage, live dead assay using Trypan blue staining on MCF-7 cells was performed. Prior to processing, a portion of the input was stained and quantified for the percentage of viable cells. Five random images were taken and averaged. The remainder culture was split into five different runs, each passing through the device three times. The outputs were subsequently stained with Trypan blue and quantified. Viability of cells at base level was at 92.8% (Fig. 4c) and the average viability after device processing was 92.9 ± 2.4% (95% CI). Using a Student’s t-test for one sample, we observed no statistical significance to assert that the mean cell viability after processing was different from the base level. Hence we affirm the device is gentle to the cells after three cycles of processing.

### Tracking of EGFR mutations and concordance measure comparing clinical samples

**Figure 5:**
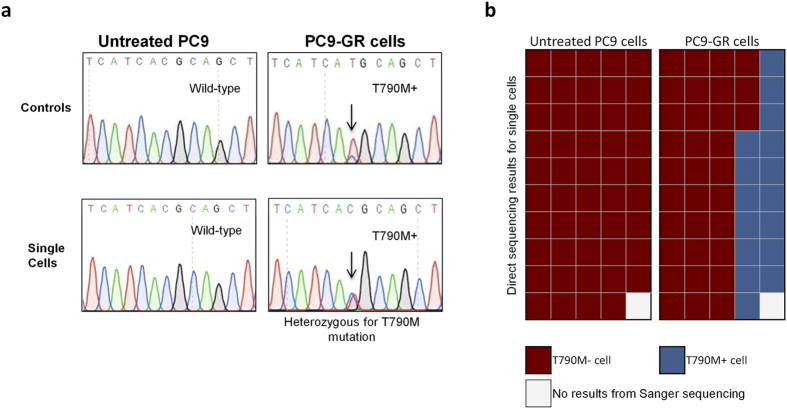
EGFR T790M (Exon 20) sequencing of PC9 cells for tracking acquired resistance after TKI treatment. (**a**) Sanger sequencing of bulk culture controls and representative single cell electropherograms selected via the device. (**b**) Quantification of 100 untreated PC9 and PC9-GR cells for the key drug resistance mutation tests via classical sequencing. Integration of downstream molecular analyses allows accurate quantification of resistant clones for monitoring purposes.

We sought to establish the capabilities for single target cell retrieval on the device and process it via conventional means of PCR and direct sequencing. Instead of pooling target cells at the recovery port, we removed each one sequentially after every ejection into 0.2 ml PCR tubes and extracted its genetic material. Conventional molecular analyses were then employed to detect the mutation of interest. As a proof of value for monitoring disease status with high sensitivity, we tracked EGFR mutation before and after acquisition of secondary resistance. A lung adenocarcinoma cell line (PC9) with an activating EGFR mutation was treated with escalating sublethal doses of gefitinib, resulting in resistant cell lines (PC9-GR), harbouring EGFR T790M mutation^53^. Primers were designed to amplify regions in exon 20, a kinase domain of the EGFR gene where the mutation resides. Results from direct sequencing of every single cell confirmed the EGFR mutations. Figure 5a,b show the collated results for a collative of 50 untreated and treated cells showcasing intercellular heterogeneity of T790M status.

Figure 5a depicts the electropherograms of pooled population of cells designated as our experimental control. Two representative electropherograms from a randomly selected cell in the untreated and PC9-GR cell groups are also shown. Results from bulk sequencing of untreated PC9 cells highlighted only wild-type EGFR while PC9-GR cells developed a small subgroup of T790M positive cells. Single cells from the same groups were then sorted and recovered using the device. For 50 single untreated cells that were isolated, one failed to yield any result from direct sequencing. This may be due to loss of material during the transfer from the device to the PCR tube or the failure of the PCR process. Success rate from cell recovery to analysis was thus shown for 49 of 50 cells, of which all did not harbor T790M mutation. These results are in line with the bulk sequencing results shown in Fig. 5a. For PC9-GR cells, 16 of 50 cells were positive for T790M mutation. Successful analysis was also performed for 49 of the 50 cells. Interestingly, our results indicated that all acquired mutant positive cells were heterozygous in nature (supplementary Fig. 3). The collective direct sequencing results from all 100 cells are illustrated in Fig. 5b. In all, the success rate for extracting information from single cells is 98% (with two failed runs). Using our device, we can actively quantify and measure proportions of PC9-GR cells that have acquired the secondary EGFR mutations after exposure to gefitinib. Our investigation yielded and confirmed PC9-GR had a positive subclone at 32% of total population.

Next, we used the same workflow for single cell extraction on seven clinical samples obtained with patient consent. The objective is to demonstrate that we can detect key driver mutations for drug response and resistance from CTCs, with a match to its primary tissue biopsies. This will be proof of its value in clinical detection which can be further expanded in the future for serial monitoring endeavors. Table 1 summarizes the background information of each sample and the results from direct sequencing of potential tumor cells retrieved from blood specimens. All samples originated from patients of stage IV NSCLC who had developed EGFR TKI resistance. Rebiopsy of accessible tumors were performed with paired blood samples drawn for CTC processing. Peripheral blood was extracted and enriched for CTCs to remove excessive WBCs. 5.9 ml to 7.5 ml of blood were used in each test for primary CTC enrichment. The input sample prior to loading onto our device had about 20,000 cells with CTCs mixed in mostly WBCs in a 10 ml buffer. We performed a secondary negative depletion of WBCs by staining for CD45 and recovered all single intact CD45 negative cells. In total, 26 potential single cells were isolated for analyses. Cell numbers from each specimen ranged from 0 to 9 cells. As an internal control, WBCs of some of the samples were also recovered for investigation.

We then performed multiplex PCR to enrich for two sites within the EGFR gene, namely T790M (in exon 20) and L858R (in exon 21). Among the seven blood samples, we have concordant data with the primary analyses for six of them (Table 1) demonstrating good sensitivity within this limited dataset (κ = 0.70 for T790M and 0.59 for L858R). Results were deemed positive if any of the isolated cells showed the intended mutation and the percentage of positive cells varied between 33% and 100%. All internal sample controls (WBCs) yielded wild-type characteristics. Our internal assay control with a healthy volunteer sample yielded no isolated CTC. Of the seven blood specimens, sample 1 recovered no CTC and hence could not be assessed for mutational analyses. In sample 2, two cells were retrieved having wild-type traits in both L858R and T790M which was representative of its tissue biopsy results. Tumor biopsies for samples 3 to 7 were a mixture of either T790M or L858R primary positives and CTCs results were entirely concordant. An added advantage over classical CTC enumeration is that we were able to determine the proportion of tumor cells harboring these mutations in blood from our analyses. This may have significant value in identifying and quantifying CTC subgroups linked to disease progression. Collating the direct sequencing results of exon 20 and 21 for all 26 cells, approximately 31% of the total cells were shown to be T790M positive (Supplementary Fig. 4), of which five were observed to be heterozygous for the mutant alleles. For L858R analyses, a total of four cells were positive for the mutation and all were homologs.

## Discussion

The microfluidic device demonstrated a systematic means of recovering single tumor cells for molecular analysis from a highly mixed population. Applying this technology to CTCs, we were able to accurately quantify EGFR mutational profiles which have significant impact on TKI drug responses in NSCLC. Similar to the study conducted by Marchetti *et al*.^37^ and Maheswaran *et al*.^38^ on CTCs, we were able to show a high concordance rate even though our patient profiles showed only low numbers of tumor cells in each sample. Thus it is important to deplete WBC prior to molecular testing to reduce possibility of false negative results. Currently, our operations to pick up single rare cells helped to potentially address key issues using liquid biopsy for monitoring and quantifying the extend of EGFR mutations due to treatment. Further automation of the system is possible with image analysis and system control. The single cell analysis which we present here will aid in revealing the dynamic nature of resistance mechanisms in solid tumors^54^.

Our approach in the microfluidic channel design is typically used in the generation of droplets^55^ for biochemical^56^ and biological studies^57^. We modified the operations such that it only focuses cells to a steady stream without forming droplets. The approach is effective to line single cells sequentially so that it can be easily interrogated. Comparing to other sheath based microfluidic particle focusing systems^58^, our method in using fluid viscosity ratios as opposed to flow-rate ratio significantly reduces the sheath fluid consumptions which is important due to our large input volume. Physical capturing chambers that are integrated alongside the main cell flow provide the means to interrogate these cells optically by immunofluorescence and morphology. As an alternative to hydrodynamic focusing, the dimensions of the fluidic channel can be reduced to 20–25 μm to achieve the same capture effect. The major drawback of this approach is that as the channel gets smaller, the fluid resistance is higher and this makes operation control more taxing. The pressure applied has to be higher to drive the flow, implying syringe pumps used has to be suited for higher torque stability. It will also be more prone to clogging when debris and cell clumps enter. This is especially critical in processing blood samples where large clumps can be expected. In our device, these clumps are actively removed without affecting the sample.

Our characterization experiments showed that we could rapidly load the device in approximately 2 s and perform cell separation via positive and/or negative selection using immunofluorescence together with the morphology of the cells. Akin to flow cytometers, we have the ability to choose to recover pooled tumor cells or as single cells. We had demonstrated samples with as little as 10 target cells could be retrieved with confidence after three processing cycles. Using conventional flow cytometry on CTCs, it was shown in one study that losses can exceed more than 50%^39^. This is important, as CTCs may exist in low numbers with certain patients’ profiles. In order to achieve the assay sensitivity required for these samples, we believe single cell analysis will overcome the variability associated with prior studies^37^,^38^. We have successfully integrated conventional PCR and Sanger sequencing to interrogate key resistance and driver mutations from single cells recovered from our system. By minimizing sample movement during isolation and applying a one step cell lysis together with PCR, the probability for the successful analysis of each cell is above 98%. The results showed we were able to accurately pinpoint the presence or absence of T790M, a common resistance mechanism in EGFR mutant lung cancer in the model PC9 cell line. In our study, we were able to measure and accurately quantify the proportion of cells acquiring the secondary EGFR mutation. While the proportion of T790M cells in circulation remains uncertain, a recent study examining TKI resistant tumor biopsies suggests that baseline allele frequency of T790M may impact on quality of 3^rd^ generation EGFR TKI^59^.

Understanding and identifying secondary resistance mechanisms are critical for treatment selection in lung cancer. In our selection of EGFR TKI resistant NSCLC patients with paired tumor rebiopsies, we detected CTCs in 6 of 7 samples with CTC counts less than 10 in each specimen. The low numbers of CTCs in NSCLC patients are a biological phenomenon observed by other studies as well^60^,^61^. Our results are in good agreement to previous findings and highlights the crucial needs of single cell analysis as the more viable option than using primary enriched CTCs in bulk cell suspensions. Without further removing all background WBCs, the concentration of mutant DNA which was as low as 1 heterozygous cell in 20,000 background WBCs or 0.0025% mutant copy would be marginalized.

In our study, we achieved 100% concordant data with the primary analyses excluding the sample without CTC. The data provides a promising outlook for single cell analysis to be routinely applied and further investigation with a larger dataset will confirm its clinical utility. Our findings established a proof of value on how analysis of single CTCs may be relevant to clinical decision-making, especially where tumor samples are unavailable and offering information relevant to clinical decision making. It also demonstrates how single CTC analysis could be used for monitoring disease progression and treatment, as blood is more readily available than tumor biopsies. Single CTC analysis provides better resolution than bulk sample testing and allows greater insights into the diverse cells in circulation. Despite the limited number of patient samples tested, our results clearly show that the CTC profiles are not homogenous across the same patient group. Within each specimen, we have shown that CTCs themselves are heterogeneous in nature and not all CTCs harbored the mutations. Combining the data from all 26 cells analyzed, we were able to accurately quantify mutant positive cells and provide insights into allelic frequencies for greater scrutiny of the disease. Data from other studies^18^ have shown that it is important to decipher the heterogeneous tumor profile. The proportion of T790M mutant allele frequencies have been found to be associated with poor PFS^62^. It is important that these cells are detected early, so as to facilitate the incorporation of therapeutic measures.

The microfluidic device we described here is a versatile tool for single cell selection from a mixed cell population. Our approach relies solely on hydrodynamic flow and physical weir structures for cell isolation and active cell recovery based on biomarker identification. The device has capacity for further throughput enhancement by increasing the number of cell chambers and added system automation. In our current study, we depleted CTCs samples entirely of WBCs and showed enhanced sensitivity for detecting mutations associated with the disease. In our clinical examples where CTC loads are scarce in blood specimens, single cell analysis is critical to ascertain the positivity in key driver mutations. Traditional bulk cellular analysis underestimates cellular heterogeneities and we envision the device will greatly aid in these situations to pick up rare cells as and when needed. We foresee the benefits in the near future of processing the single cells collected through our system for a larger genetic panel study using next generation sequencing to better understand CTC heterogeneity. This real-time CTC analysis will bring much needed important information not only on the possible development of drug resistance over the course of anticancer therapy, but also for better personalized treatment decisions for cancer patients.

## Methods

### Device fabrication

Figures 1 and 2a shows the schematics of the design layout. The device was made using soft lithography that had been previously described^63^. Briefly, the process is as follows. The photomask shown in Fig. 2a was drawn using AutoCAD 2011 (Autodesk, Inc., San Rafael, CA, USA). The master mold for replica molding was done using two layers of SU8 (MicroChem Corp., MA, USA) photo patterning. The first layer of 2 μm thickness was fabricated by spin coating SU8-2002 (500 rpm 5 s 100 rpm/s; 2000 rpm 30 s 300 rpm/s) on a 4-inch wafer for the reverse weir structures. The second layer was produced by spin coating SU8-2025 (500 rpm 5 s 100 rpm/s; 3000 rpm 30 s 300 rpm/s) on the existing patterns to create the cell chambers at the weir structures. The mold was completed after a hard bake at 200 °C for 15 minutes.

Polydimethylsiloxane (PDMS) in a base to curer ratio of 10:1 (Sylgard 184, Dow Corning Corp, MI, USA) was casted on the master mold fabricated using soft lithography methods. The PDMS mixture was then degassed in a desiccator for one hour. Following the removal of air bubbles, the mixture was cured in an oven at 70 °C for 1 hour and cut into 3.5 cm × 2.5 cm sizes. All fluidic access holes were made with a 0.5 mm biopsy punch while the collection port was made with 2.0 mm biopsy punch. The completed PDMS device was bonded to the glass slide by plasma-activated bonding (FemtoScience CUTE-B plasma system, Korea) by holding vacuum for 5 minutes followed by a 2 minutes plasma treatment at 80% power output in the presence of oxygen gas.

### Buffer preparation and system operation

Based on viscosity values of Glycerol (ThermoFisher Scientific, MA, USA) and Polyethylene glycol (PEG) 35000 (Sigma-Aldrich, Singapore), we determined the optimal flow rate and flow ratio by observing the focused stream size. All reagents were filtered (0.20 μm Minisart syringe filter) and diluted with deionized water to get the desired concentration. Food dyes were diluted 1:200 to visualize and distinguish cell flow from sheath flow in the device. All control ports were kept closed while the waste outlet was opened before running the micro-device. The flow control was operated using a Syringe Pump (Chemyx Fusion 200 Classic, TX, USA) and controlled with a custom-built USB electronic controller unit incorporated to a PC. Image acquisition was performed with a CMOS Camera (DCC1645C, Thorlabs, NJ, USA) mounted on the microscope (Olympus BX61, Japan).

Efficient single cell capture was crucial in establishing rare cell isolation and throughput. Polystyrene beads of 15 μm (flow cytometry size calibration kit, Life technologies CA, USA) and 20 μm (Polysciences, Inc., PA, USA) were used to characterize the performance of the microfluidic device. After the cell and sheath flows within the micro-device stabilized, all 10 control ports were opened together. The capture of polystyrene beads in the cell chambers were recorded using a high speed camera (Photron Fastcam 1024PCI, CA, USA) mounted on the microscope (Olympus BX61) with the following settings: 1000 fps, manual mode, 40% of total frame prior to endless mode trigger, 512 × 512 resolutions (total recorded time of 6.4 s). Video recording was initiated when the first bead was seen captured in the activated control port.

### Cancer cell preparation for cell separation and T790M monitoring

WBCs were isolated from whole blood using Lymphocyte Separation Medium (LSM) (Mediatech Inc., VA, USA) and stained with Anti-Human CD45 PE (eBioscience, Inc., CA, USA). Blood samples used in all spiked experiments were obtained from various healthy donors recruited in the study with consent given. MCF-7 cancer cells were used in initial characterization experiments and stained with Anti-Cytokeratin (CK3-6H5)-FITC, (Miltenyi Biotec, Singapore) following a permeabilization step using 0.1% Triton X (Sigma Aldrich, Singapore). For maintaining the cell line, culture was done with DMEM medium supplemented with 10% FBS and 1% penicillin streptomycin (pen strep) in T25 flasks. Cultures were passaged when they reached 90% confluence. Both cell populations (WBCs and MCF-7) were subsequently stained with Hoechst 33342, Trihydrochloride, Trihydrate (Life Technologies, CA, USA). Cancer cells were released into the recovery port while WBCs were released into the waste port by clamping the exit of each unused port in the collection/waste during cell selection. Cells were transferred onto a glass slide and imaged on the microscope. Trypan blue (Thermo Fisher, USA) staining was performed to ascertain the number of viable cells before and after device processing. Final concentration of 0.1% Trypan blue was used and percentage of viable cells was calculated using equation 1.


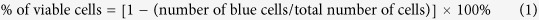


For T790M tracking trials using single cells, untreated PC9 cells were cultured in T25 flasks with RPMI 1640 medium (10% FBS, 1% Pen Strep). Generation of the resistant cell line was described previously^64^ with slight protocol modifications. PC9-GR cells were exposed to gefitinib in increasing doses from 0.1 μM up to 6.4 μM and cultured over an extended period of three months. Single cells were sequentially recovered in the collection port and aliquot into a PCR tube containing lysis solution (Ambion^®^ Single Cell Lysis Kit, Life Technologies, CA, USA). Extraction of DNA was achieved following manufacturer’s recommendations without adding the DNase. PCR mix containing primers for T790M was added thereafter.

Forward: 5′-TGTAAAACGACGGCCAGTCCATGAGTACGTATTTTGAAACTC-3′

Reverse: 5′-CAGGAAACAGCTATGACCCATATCCCCATGGCAAACTCTTGC-3′

Thermocycling conditions were as follows: 94 °C for 2 minutes, forty cycles of 94 °C for 15 s, 56 °C for 30 s, 72 °C for 45s, one cycle of 72 °C for 7 minutes and hold at 4 °C. Amplicons were purified using Agencourt Ampure XP (Beckman Coulter, CA, USA) following manufacturer’s processes. Sanger sequencing was outsourced to Axil Scientific, Singapore.

### Clinical samples processing

Blood samples from stage IV NSCLC patients and healthy donors were obtained with informed consent in accordance with the approved procedures under the institutional review board (IRB) guidelines. The project was approved by the Singhealth Centralised Institutional Review Board (CIRB).

Analysis of the primary tumor was performed as part of the routine procedure during patient recruitment. Sample workflow for single cell capture is illustrated in supplementary Fig. 5 with total processing time of 7.5 hours. Blood samples of 7.5 ml were extracted from each patient if possible. Primary CTC enrichment was carried out via the ClearCell FX system (Clearbridge Biomedics, Singapore) at the Singapore General Hospital. The mixed cell population was then stained with Anti-Human CD45 PE (eBioscience, Inc., CA, USA) and further depleted of WBCs using our device and recovered as single cells in PCR tubes. The additional primer pair for L858R is as follows.

Forward: 5′- CTAACGTTCGCCAGCCATAAGTCC-3′

Reverse: 5′- GCTGCGAGCTCACCCAGAATGTCTGG-3′

Amplicons were sent for direct sequencing after purification. Results were single blinded and compiled after analyzing the direct sequencing results of all isolated single cells.

### Statistical analysis

Student’s t-tests were performed on the beads capture efficiency and potential cell damages using the device. Categorical data were presented as mean ± SEM and Cohen’s kappa was used to deduce the significance between results of primary tissue analysis and CTCs. All statistical analysis was calculated using the Prism software (Graphpad, San Diego, USA).

## Additional Information

**How to cite this article**: Yeo, T. *et al*. Microfluidic enrichment for the single cell analysis of circulating tumor cells. *Sci. Rep.* **6**, 22076; doi: 10.1038/srep22076 (2016).

## Supplementary Material

Supplementary Information

Supplementary Movie S1

Supplementary Movie S2

## Figures and Tables

**Table 1:** CTC enumeration and EGFR mutational analyses from late stage NSCLC patients.

	Patient Details			Primary Mutational Analyses		CTC single cell analyses				
	Tumor site	Stage	Gender	T790M	L858R	CTC enumeration	Blood volume tested (ml)	T790M	L858R	Concordant
1	NSCLC	IV	F	+	+	0	5.9	NA	NA	NA
2	NSCLC	IV	F	—	—	2	7.5	(−) 0%	(−) 0%	YES
3	NSCLC	IV	F	+	—	4	7.5	(+) 50%	(−) 0%	YES
4	NSCLC	IV	F	+	—	9	6	(+) 33%	(−) 0%	YES
5	NSCLC	IV	M	+	—	2	7.5	(+) 100%	(−) 0%	YES
6	NSCLC	IV	F	—	+	8	7.5	(−) 0%	(+) 53%	YES
7	NSCLC	IV	F	+	—	1	7.5	(+) 100%	(−) 0%	YES
	Healthy Volunteer	NA	M	NA	NA	0	7.5	NA	NA	NA
							κ*	0.70	0.59	

Primary mutational analyses derived from tumor re-biopsies at point of resistance; CTC: Circulating tumor cell; CTC enumeration is achieved by analyzing CD45-negative cells recovered from the system. The proportion(%) of positive mutants is calculated by taking the ratio of cells showing a mutant signature over the total number of CTCs for that specimen.

^*^Cohen’s kappa was calculated with patient 1 being negative for both tested sites.
