# Supplementary material for: Microfluidic enrichment for the single cell analysis of circulating tumor cells
Source: Sci Rep. 2016 Feb 29;6:22076. doi: 10.1038/srep22076 (PMC4770429; doi:10.1038/srep22076)
Supplement: Supplementary Information [file srep22076-s1.doc]

**Supplementary Information**

Microfluidic enrichment for the single cell analysis of EGFR mutations in circulating tumor cells

Trifanny Yeo^1#^, Tan Swee Jin^1#^*,Lim Chew Leng^2^, Dawn Lau Ping Xi^3^, Chua Yong Wei^4^, Sai Sakktee Krisna^3^, Gopal Iyer^3^, Tan Gek San^6^, Tony Lim Kiat Hon^6^, Daniel Tan S.W.^3-5^, Wan-Teck Lim^4^*, Lim Chwee Teck^7,8^*


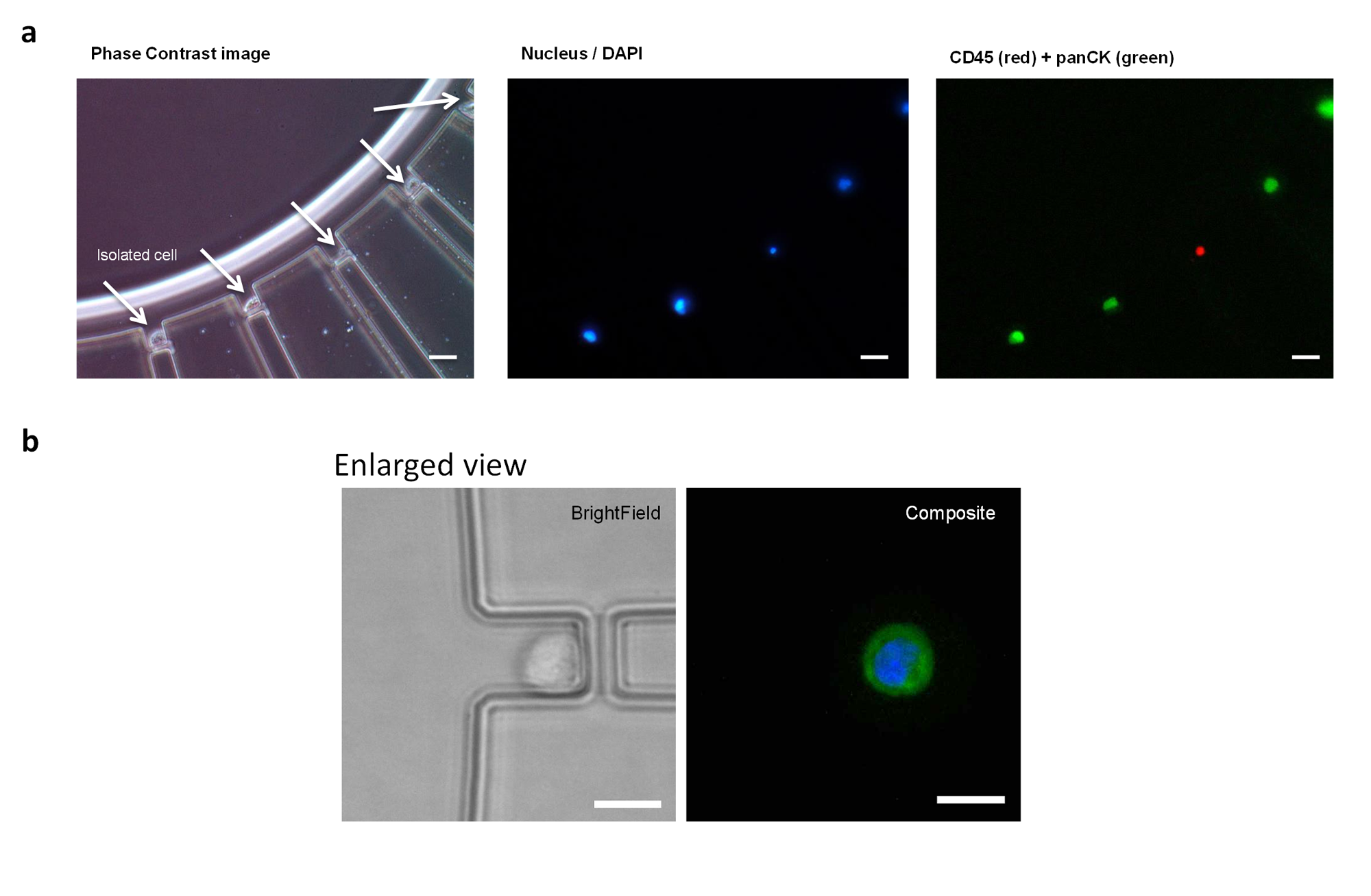


**Supplementary Figure 1**. Active cell selection from a spiked sample control showing the straightforward process of separation. WBCs marked by CD45 positivity (red cells) are discarded. (B) Image of captured cell taken at 40χ magnification. Scale bar represents 20 μm.


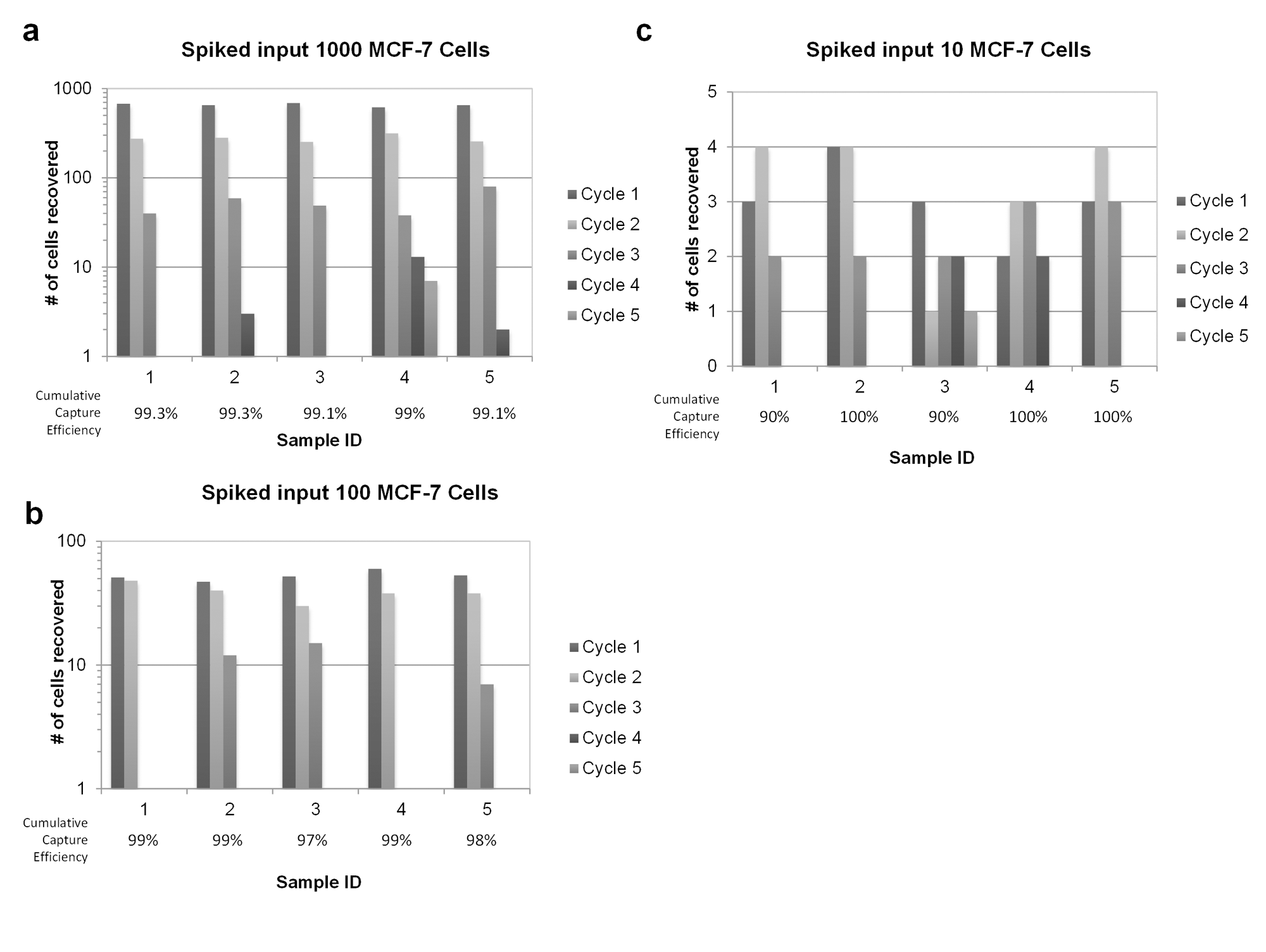


**Supplementary Figure 2**. Cell recovery efficiency at different input conditions at different cycles of processing in the device. (a) Spiked samples of 1000 MCF-7 cells with five independent experiments. Maximum recovery of 99.3% is achieved. (b) Input concentration of 100 MCF-7 cells with maximum recovery of 99%. (c) Input concentration of 10 MCF-7 cells with maxima efficiency of 100%.


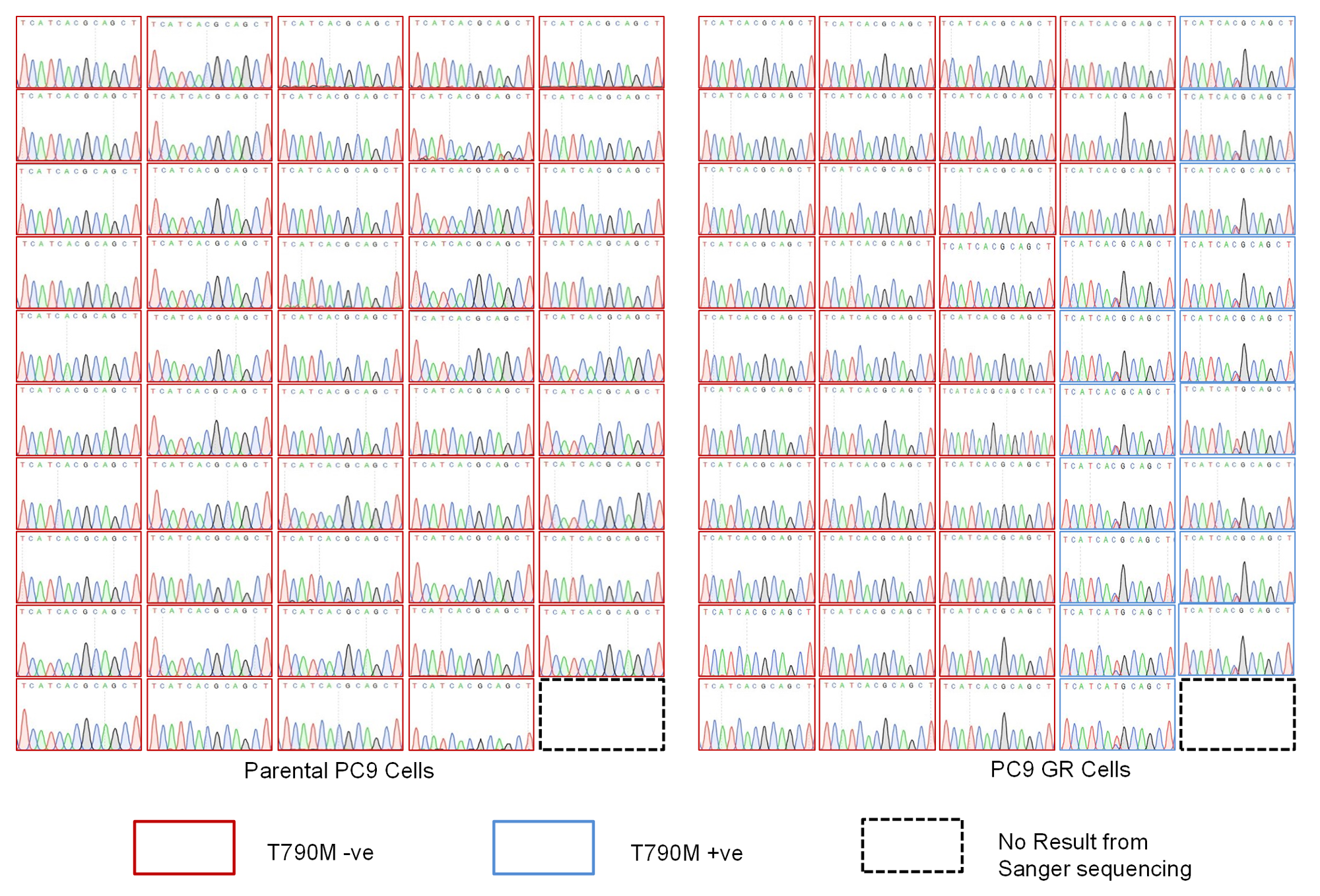


**Supplementary figure 3**. Monitoring of PC9 cells via classical Sanger sequencing for T790M mutations. (a) 50 isolated untreated cells that show all wildtype characteristics. (b) PC9-GR cells isolated via our device for comparison showing acquired mutational profiles for a small subpopulation of cells.


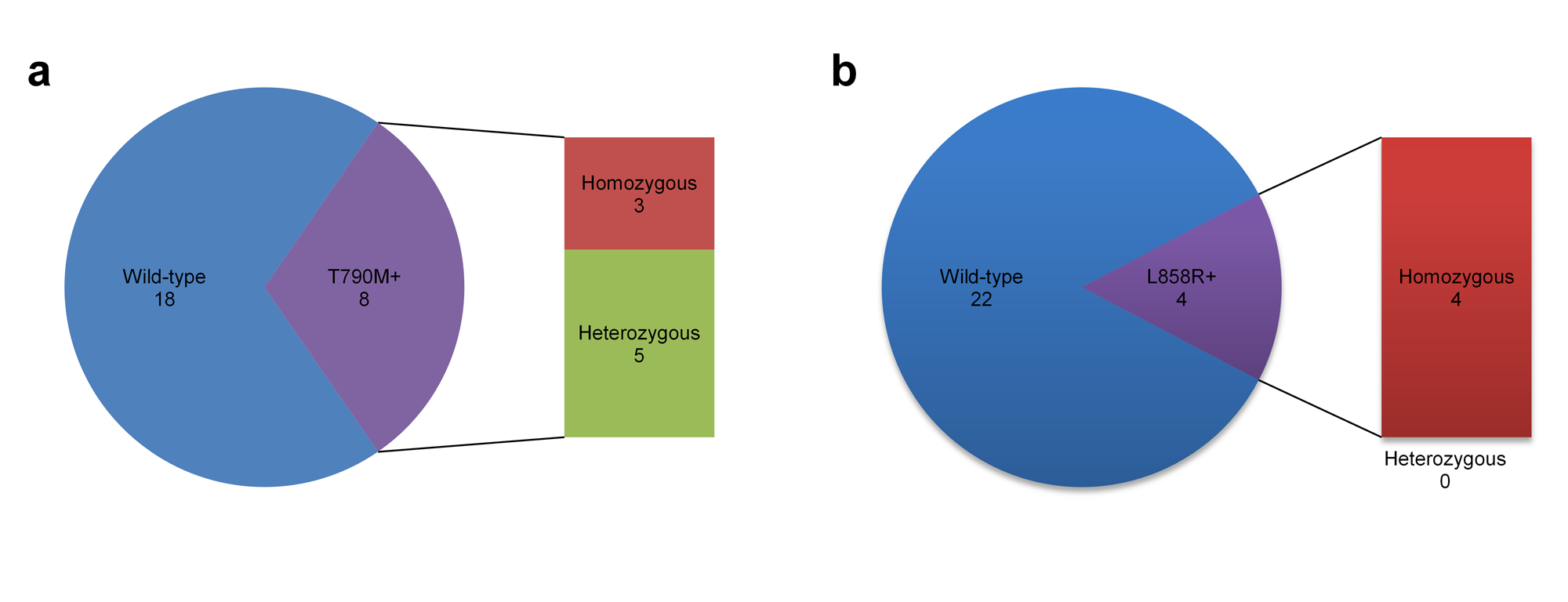


**Supplementary figure 4**. Distribution of cells harboring the target EGFR mutations. (a) Eight cells of 26 isolated single cells from the clinical samples show T790M positivity. Direct sequencing further shows 3 are homozygous. (b) 4 cells show L858R positivity and all are homozygous from direct sequencing.


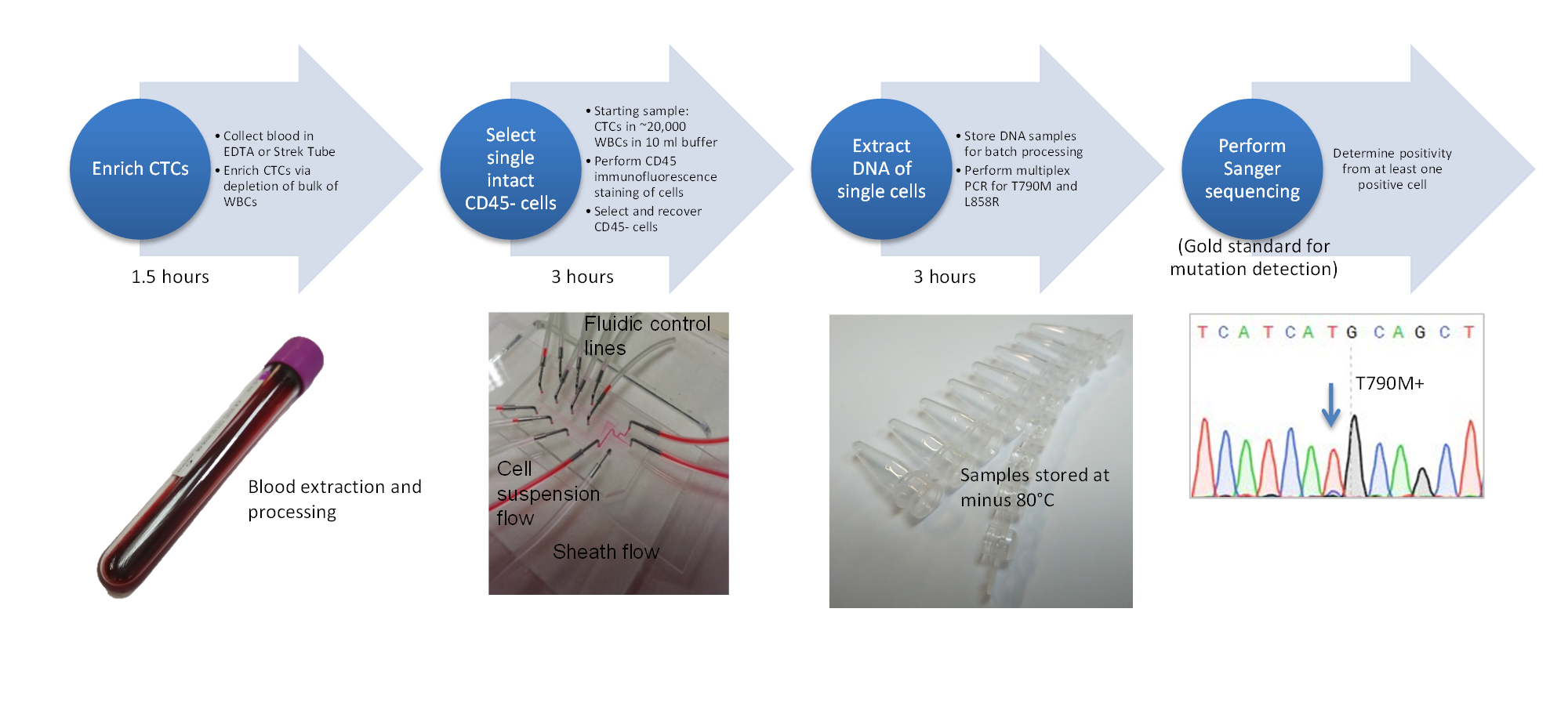


**Supplementary figure 5**. Clinical specimen processing workflow using classical sequencing to readout the mutational states for each single cell processed from the device.


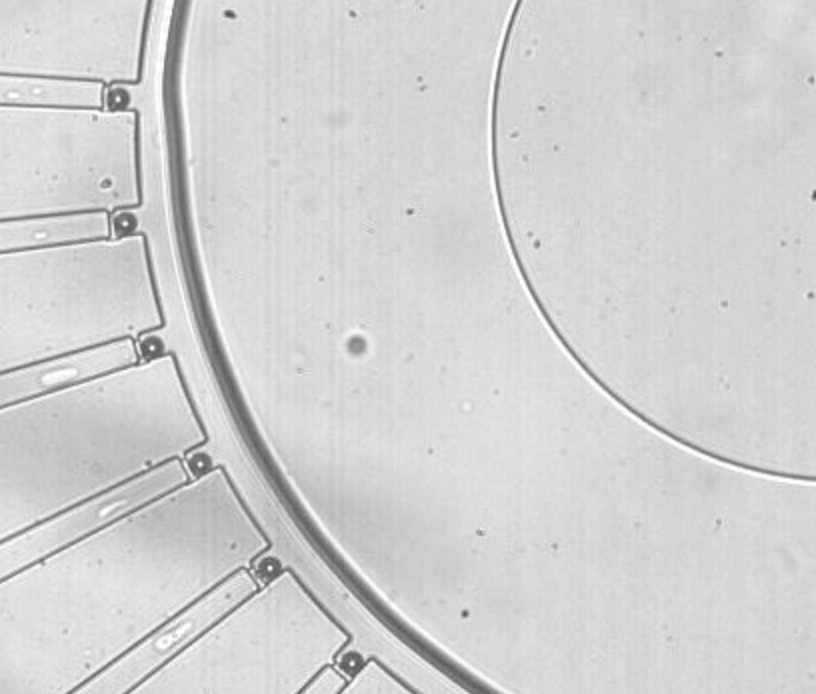


Isolated bead

Movie S1. Single particle isolation efficacy measurements using polystyrene beads


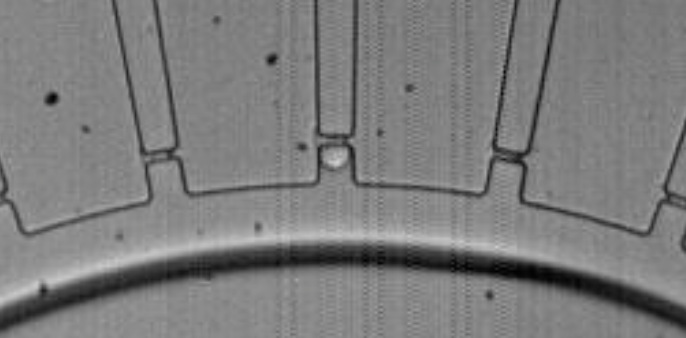


MCF-7

Movie S2. Selective trapping and recovery of single MCF-7 cell
